# Disordered Regions of Mixed Lineage Leukemia 4 (MLL4) Protein Are Capable of RNA Binding

**DOI:** 10.3390/ijms19113478

**Published:** 2018-11-05

**Authors:** Beáta Szabó, Nikoletta Murvai, Rawan Abukhairan, Éva Schád, József Kardos, Bálint Szeder, László Buday, Ágnes Tantos

**Affiliations:** 1 Institute of Enzymology, Research Centre for Natural Sciences, Hungarian Academy of Sciences, H-1117 Budapest, Hungary; szabo.beata@ttk.mta.hu (B.S.); murvai.nikoletta@ttk.mta.hu (N.M.); rawan.abukhairan@ttk.mta.hu (R.A.); schad.eva@ttk.mta.hu (E.S.); szeder.balint@ttk.mta.hu (B.S.); buday.laszlo@ttk.mta.hu (L.B.); 2 ELTE NAP Neuroimmunology Research Group, Department of Biochemistry, Eötvös Loránd University, H-1117 Budapest, Hungary; kardos@elte.hu

**Keywords:** MLL proteins, MLL4, lncRNA, HOTAIR, MEG3, leukemia, histone lysine methyltransferase, RNA binding, intrinsically disordered protein

## Abstract

Long non-coding RNAs (lncRNAs) are emerging as important regulators of cellular processes and are extensively involved in the development of different cancers; including leukemias. As one of the accepted methods of lncRNA function is affecting chromatin structure; lncRNA binding has been shown for different chromatin modifiers. Histone lysine methyltransferases (HKMTs) are also subject of lncRNA regulation as demonstrated for example in the case of Polycomb Repressive Complex 2 (PRC2). Mixed Lineage Leukemia (MLL) proteins that catalyze the methylation of H3K4 have been implicated in several different cancers; yet many details of their regulation and targeting remain elusive. In this work we explored the RNA binding capability of two; so far uncharacterized regions of MLL4; with the aim of shedding light to the existence of possible regulatory lncRNA interactions of the protein. We demonstrated that both regions; one that contains a predicted RNA binding sequence and one that does not; are capable of binding to different RNA constructs in vitro. To our knowledge, these findings are the first to indicate that an MLL protein itself is capable of lncRNA binding.

## 1. Introduction

Long non-coding RNAs (lncRNAs) are transcribed RNA molecules longer than 200 nucleotides that do not code for translated proteins. The human genome is estimated to code for about 58,000 lncRNAs [1], that are being more and more recognized as central players in a plethora of biological processes. They can act as flexible scaffolds providing binding platforms for different proteins, they can interfere with other endogenous RNAs acting as microRNA “sponges” and they can modify chromatin state [2], thus regulating the expression of various proteins. LncRNAs have also been shown to play a role in several layers of epigenetic regulation: they are involved in DNA methylation and demethylation, they can modify chromatin conformation through binding to remodelers [3] and many of them interact with histone modifier enzyme complexes such as PRC2, coREST or SMCX [4].

The physiological processes where lncRNA regulation have been suggested involve cell cycle regulation, epithelial mesenchymal transition (EMT) [5], cancer progression [6] and maintenance of cancer stem cells [5], hypoxia [7] and leukemia [8].

Various lncRNAs are shown to have altered expression levels in different leukemias, resulting in a crucial influence on cellular transformation [9], chromosomal translocation [10], apoptosis [11] and on drug resistance [12]. Accumulating evidence regarding the involvement of lncRNAs in leukemic processes prompted the suggestion to use them as prognostic and classification factors. It was found that lncRNA expression has prognostic value in AML patients [13] and multiple pathways were involved in lncRNA expression, including chromosome organization and trans-membrane receptor protein tyrosine kinase signalling pathway.

As lncRNAs are also considered valuable drug targets, it is essential that the molecular details of their functions are uncovered.

Polycomb repressive complex (PRC2) is the most studied histone modifier that relies on lncRNA binding in its function, being able to bind several lncRNAs including HOTAIR, Xist, RepA, Braveheart, MALAT1 and MEG3 [14]. In vitro experiments revealed that not only EZH2, but other PRC2 subunits are also capable of lncRNA binding [15], thus providing a pattern of binding regions distributed along the surface of the complex. Even though there remain open questions regarding the specificity of the RNA binding by PRC2 [16], it is widely accepted that lncRNA binding plays a defining role in PRC2 targeting and the ensuing gene silencing [14]. It is interesting to note that despite the numerous experimental results that show EZH2 to be an RNA binding protein, it cannot be found in databases that list RNA binding proteins, furthermore no RNA binding site is predicted to be located in the region that is shown to be responsible for the RNA-protein interaction [17].

Apart from PRC2, other histone lysine methyltransferases (HKMTs) or HKMT complex components also appear to bind lncRNAs with a relevant physiological outcome.

LncRNA EZR-AS1 enhances EZR expression through recruiting SMYD2 to the upstream region of its promoter region and elevating the activating H3K4 methylation [18].

G9a interacts with lncRNA PARTICLE to regulate MATA2 expression upon mild irradiation [19]. The interaction was shown using ChIP assay and apart from G9a, the PRC2 subunit Suz12 was also pulled down. In a later experiment, it was found that PARTICLE can also interact with DNA methylase DNMT1 and that it increases H3K27 methylation as well as EZH2 expression. It was suggested that PARTICLE may serve as a functional platform that enables the specific targeting of chromatin modifiers, such as PRC2 [20].

WDR5, a component of the MLL1-4 and SET1a/1b complexes was proven to interact with lncRNAs NeST and HOTTIP with an effect on microbial susceptibility through the enhancement of interferon-γ expression [21]. Further investigation of the WDR5-HOTTIP interaction led to the recognition that lncRNA binding by WDR5 is essential in maintaining embryonic stem cell pluripotency [22]. However, not this work nor any previous studies investigated the possibility that the enzymatic component of the methyltransferase complex may also be capable of lncRNA binding.

The family of mammalian MLL (Mixed Lineage Leukemia) proteins consist of Set1a, Set1b and four MLL proteins, MLL1, MLL2, MLL3 and MLL4. They work in COMPASS-like complexes and catalyze H3K4 mono-, di- or tri-methylation, each complex having different specificity and methylase activity [23]. MLL3 and MLL4 are responsible for the monomethylation of H3K4 at enhancer regions [24] and has been linked to a high number of different cancers. Properly functioning MLL3 and MLL4 act as tumor suppressors [23], therefore mutations affecting their activity or stability can result in cancer development. Despite their central role in several types of cancers, many open questions regarding the regulation of the activity and the targeting of the MLL complexes remain unanswered. The exact molecular details of how MLL3 and MLL4 are targeting enhancer regions [23] as well as the specific molecular effects of the interactions of their different regulatory domains [25] are largely unknown. It is also worth noting that the known structured domains represent only 15–21% of the sequences of MLL proteins, leaving the vast majority of these proteins uncharacterized both structurally and functionally.

In a previous work [26] we suggested that the disordered regions of HKMTs may harbor so far unrecognized interaction sites, adding more layers of the regulation of their activity. Based on the observation that many lncRNAs are involved in processes governed by HKMTs, we hypothesized that lncRNA binding might be one of the functions of these regions.

Since multiple evidence point in the direction that leukemic processes are fundamentally affected by lncRNAs and MLL complexes are involved in this regulation, we concentrated on MLL proteins. Taken the analogy of the PRC2 complex, where more than one complex subunits are capable of lncRNA binding, we aimed at testing the ability of MLL4 to bind different RNA molecules.

## 2. Results

### 2.1. In Silico Analysis of the RNA Binding Capacity of MLL Proteins

As a first step, we mapped the predicted RNA binding motifs on the sequence of four MLL proteins. We used DisoRDPbind, an RNA interaction prediction tool specifically designed to find RNA interaction sites in the disordered regions of proteins. Results shown in Table 1 indicate that all MLL proteins contain several putative RNA interaction motifs in their disordered regions. These regions are found at various positions in the proteins and vary in length from a couple of amino acids to almost a hundred residues, suggesting that RNA binding might be a common feature in MLL proteins.

**Figure 1:**
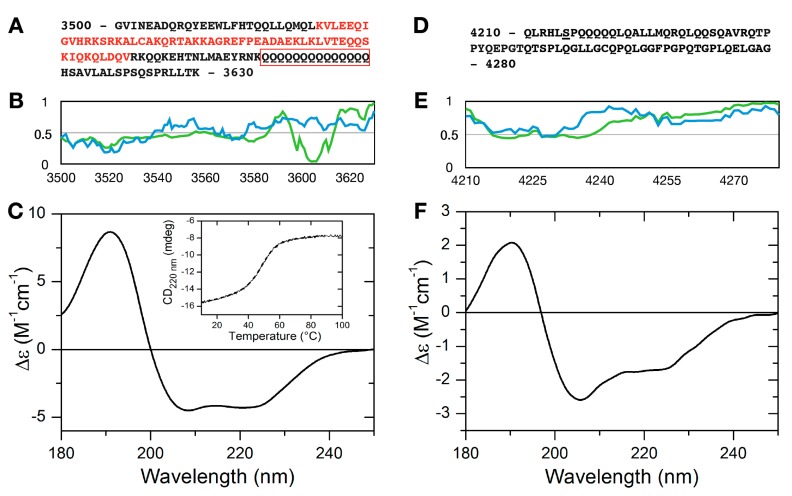
Structural characterization of the MLL4 regions. Sequences of MLL4_3500–3630_ (**A**) and MLL4_4210–4280_ (**D**). Predicted RNA binding region is indicated by red letters and the polyQ stretch is framed with red. IUPRed (blue) and Anchor (green) prediction of MLL4_3500–3630_ (**B**) and MLL4_4210–4280_ (**E**). Residues having an IUPred score above 0.5 are considered to be disordered, while residues with an Anchor score below 0.5 constitute predicted binding sites. Far-UV CD spectra of MLL4_3500–3630_ (**C**) and MLL4_4210–4280_ (**F**). Inset: temperature-dependent changes in the structure of MLL4_3500–3630_ as observed by monitoring the changes in the absorbance at 220 nm.

A comparison with our earlier studies [26] revealed that two conserved disordered binding sites (residues 3537–3545 and 3560–3567) reside within one of the predicted RNA binding regions (residues 3526–3581, Figure 1A) of MLL4, underlining the reliability of the predictions. This region also harbors several cancer-related point mutations, two of them corresponding to a predicted binding site at positions 3560 (D-N) and 3561 (A-D). All these evidences point to the physiological importance of this protein region, making its structural and functional study worthwhile. ANCHOR prediction [27] shows that within the C-terminal border of the predicted RNA binding region there is a region with a strong tendency of the protein chain to form protein-protein interactions (residues 3597–3613, Figure 1A) that corresponds to a run of 14 glutamine residues. Since polyQ repeats in RNA binding proteins have been linked to protein-RNA droplet formation [28], this raises the intriguing possibility of granule formation potency of this segment. Therefore, we chose to test the RNA binding capacity of the MLL4 region between residues 3500–3630 (Figure 1A). As an internal control, another disordered region with no predicted RNA or protein binding sites was selected between residues 4210–4280 of MLL4 (Figure 1D).

As for binding RNAs, we opted to test two different lncRNA constructs, both having been reported to play a role in leukemias. The first is HOTAIR, that has the ability to bind EZH2 (PRC2). The 5’ 300 nucleotides of HOTAIR are thought to mediate its binding to PRC2 complex subunits, but the latest annotation in the NCBI database contains an additional 140 bases at the beginning of HOTAIR sequence, compared to the one reported earlier. Therefore, we prepared the longer version (HOTAIR_440_) that encompasses the 300 nucleotides already known to be involved in protein-RNA interactions and also the nucleotides that has not been studied yet. Since there is no information available about the region of MEG3 that is able to bind proteins, we used the full length MEG3 for our experiments.

### 2.2. Secondary Structure of MLL4_3500–3630_ and MLL4_4210–4280_

Disorder prediction profiles (Figure 1B,E) indicated that both protein regions have a significant disorder tendency. Disorder profile of MLL4_3500–3630_ indicates a rather ambiguous disorder state, with prediction scores fluctuating around the 0.5 limit between ordered and disordered states. This disorder prediction might indicate a disordered region that has an elevated tendency to fold or a relatively unstable folded segment as well. Far-UV CD measurements revealed that MLL4_3500–3630_ has a helical structure in isolation (Figure 1C). The CD spectrum of this region of MLL4 showed a typical alpha helical conformation with a pronounced double minimum at 208 and 220 nm. Secondary structure content calculation using the BeStSel algorithm [29,30] gave an α-helix content of ~36.2%, while another ~36% of the secondary structure content was characterized as “Others”, which mainly corresponds to the disordered structure. Thermal unfolding of the observed helical structure was followed by gradually heating the sample to 100 °C while recording the absorbance at 220 nm (Figure 1C inset). The melting curve indicated a cooperative unfolding of the structure with a melting point of 48 °C. The CD spectrum of the thermal denatured state is shown in Supplementary Figure S1, demonstrating a complete loss of structure at high temperatures.

MLL4_4210–4280_ has a more pronounced disorder tendency, as demonstrated by the IUPred profile and is devoid of any predicted ANCHOR binding sites (Figure 1E). Its sequence contains a significant portion of glutamines (Figure 1D), but it does not contain Q stretches longer than 4 residues. Far-UV CD measurements confirmed the disorder predictions, indicating that the protein is mostly disordered in solution, with a considerable α-helical tendency. Secondary structure calculations gave a result of 16% α-helix and ~45% “Others” content, underlining that this segment of MLL4 is not fully disordered and contrary to interaction site predictions, might be involved in molecular recognition.

### 2.3. RNA Binding of MLL4_3500–3630_ and MLL4_4210–4280_

**Figure 2:**
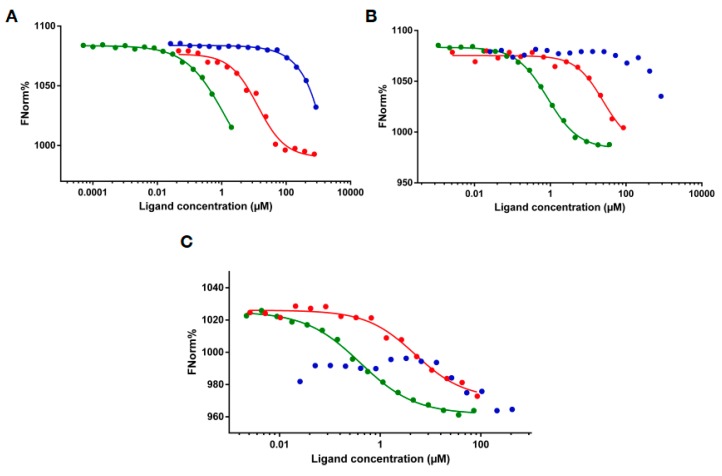
RNA binding detected by microscale thermophoresis. MST binding curves of MLL4_3500–3630_ (green), MLL4_4210–4280_ (red) and thymosin beta 4 (blue) to different RNAs: HOTAIR_440_ (**A**), MEG3 (**B**) and 50 nt RNA (**C**).

Microscale thermophoresis measurements were performed to characterize the RNA binding of the expressed protein regions. We used two lncRNA constructs, HOTAIR_440_, a segment of HOTAIR that contains the region involved in binding to EZH2 [31], MEG3, a lncRNA involved in leukemias [32] and a 50 nt long RNA with random nucleotide sequence. Contradicting to the lack of predicted binding sites, MLL4_4210–4280_ showed a relatively strong binding to HOTAIR_440_ with an apparent Kd of 13.05 μM (Figure 2A), while the negative control Thymosin beta 4 (Tβ4) did not bind to the RNA, showing any sign of interaction at only the highest concentrations applied.

**Figure 3:**
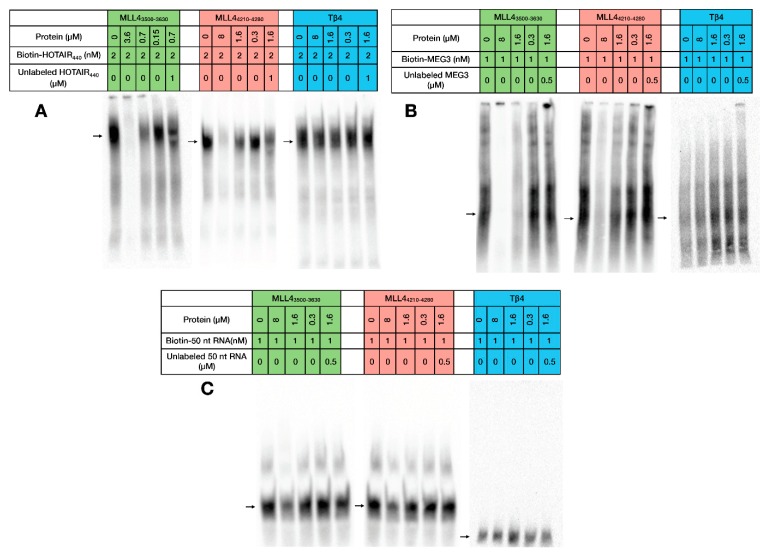
Electrophoretic Mobility Shift Assay. Interaction of MLL4_3500–3630_, MLL4_4210–4280_ and Tβ4 with HOTAIR_440_ (**A**), MEG3 (**B**) and 50 nt RNA (**C**). For easier understanding, the coloring scheme of Figure 2 is followed (MLL4_3500–3630_: green MLL4_4210–4280_: red, Tβ4: blue). Free RNA is indicated by arrows.

In the case of MLL4_3500–3630_, saturation of the reaction could not be reached because of marked aggregation above 1:20 RNA:protein ratio (Supplementary Figure S2) but using the T-jump values of the MST measurement (Supplementary Figure S3) an approximate binding constant of 0.1 μM could be determined. The appearance of large particles in the solution, generally considered to be aggregates, is indicated by a “wavy” MST curve and a randomly fluctuating normalized fluorescent percentage as shown on Supplementary Figures S2 and S5. The observed aggregation was dependent on the RNA species, since it was not seen with either of the other tested RNAs (Figure 3B,C), or with a shorter, 300 nt long HOTAIR construct (Supplementary Figure S4). The HOTAIR_300_ construct overlaps with HOTAIR_440_ in the 3′ 300 nucleotides but lacks the first 140 nucleotides of the latter. This shorter HOTAIR construct bound to MLL4_3500–3630_ with a Kd of 0.97 μM, with no sign of irregular behavior. Centrifugation (15 min at 13,000× *g*) of the samples resulted in the loss of fluorescent signal in a protein concentration-dependent manner (Supplementary Figure S5), indicating a formation of structures containing both RNA and protein. Such phenomenon was not observed with MLL4_4210–4280_, or Tβ4 upon mixing them with HOTAIR_440_, even at significantly higher protein concentrations than MLL4_3500–3630_. Also, MLL4_3500–3630_ did not show aggregation-prone behavior in the absence of RNA.

As we experienced no anomaly in the behavior of MLL4_3500–3630_ when titrated to MEG3, determination of a binding constant was straightforward for this interaction. As shown in Figure 2B, affinity to MEG3 of this region of MLL4 was higher than that of MLL4_4210–4280_. The Kd of MLL4_3500–3630_ binding to MEG3 was calculated to be 0.722 μM, while Kd calculation for MLL4_4210–4280_ was not reliable since saturation of the reaction could not be reached throughout the protein concentration range tested. Tβ4 did not show significant affinity to MEG3, resulting in a failure of binding curve fitting.

To check for any specificity of binding that the expressed MLL4 regions may possess, we also tested a physiologically non-relevant 50 nt RNA construct. Binding curves presented in Figure 2C indicate that both MLL4_3500–3630_ and MLL4_4210–4280_ are capable of binding to this RNA species, but with a remarkably lower affinity than to the lncRNA constructs, while Tβ4 could not bind to it at all. The extended shape of the binding curve and the absence of saturation in the case of both MLL4 constructs indicate weak binding that resulted in an inability to reliably determine the binding constants. Nevertheless, MLL4_3500–3630_ still displayed a stronger affinity towards the RNA than MLL4_4210–4280_.

Electrophoretic Mobility Shift Assay (EMSA) experiments confirmed the findings of the MST measurements (Figure 3) as both MLL4 regions caused a significant change in RNA mobility in the case of HOTAIR_440_ and MEG3 (Figure 3A,B) RNAs. This shift was drastically less pronounced with the 50 nt RNA sample (Figure 3C), resulting only in a minor weakening of the RNA signal in the lane with the highest protein concentration. This observation corresponds to the outcome of the MST experiments, underlining the existence of a certain level of specificity in the RNA recognition by these two MLL4 regions. The negative control Tβ4 failed to cause any visible change in the RNA mobility, indicating a lack of interaction with any of the tested RNAs. Competitive RNA binding (Figure 3, compare the 3rd and 5th lanes) demonstrated that the observed shift in mobility was indeed a result of RNA-protein interaction, since the shift could be prevented at least to some extent by adding excess unlabeled RNA to the reaction mixtures.

The anomalous behavior of the MLL4_3500–3630_:HOTAIR_440_ interaction observed in MST was seen in the EMSA experiments as well, since at high protein:RNA ratios the samples obtained a highly viscous quality and completely remained in the wells during the electrophoretic run. Successful experiments could only be carried out by lowering the applied protein concentration, but the interaction was clearly observable even under these circumstances.

In all of the tested interactions, MLL4_3500–3630_, which contains a predicted RNA binding region presented higher affinities to RNAs than the other MLL4 segment, indicating the validity of the prediction. On the other hand, binding of MLL4_4210–4280_ could also be detected in all cases, raising the possibility of the existence of RNA binding sequences differing from the already described interaction motifs. EZH2, a known RNA binding HKTM also interacts with RNAs through a region [17] that has no recognizable RNA binding sequence, emphasizing our lack of complete knowledge of the sequential determinants of protein-RNA interactions.

## 3. Discussion

**Figure 4:**
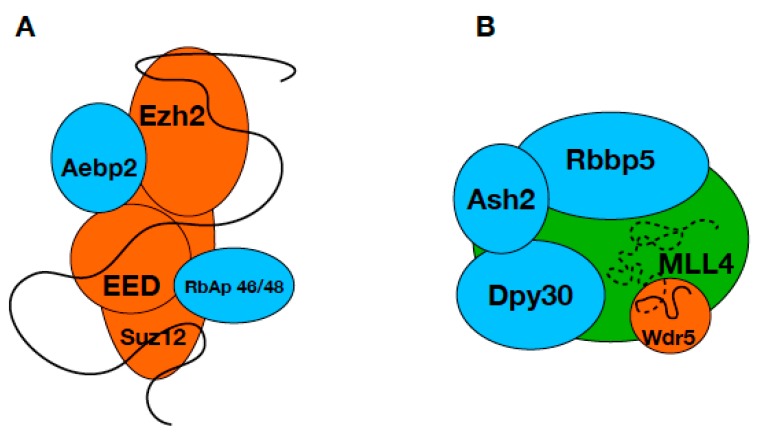
lncRNA binding of PRC2 and MLL4/COMPASS complex. Schematic representation of the PRC2 (**A**) and MLL4/COMPASS (**B**) complexes, where the known RNA binding subunits are shown in orange and the suggested lncRNA binding subunit MLL4 is green. Subunits currently not known to be involved in lncRNA binding are blue and the lncRNA is represented by a black line. Suggested lncRNA-MLL4 interaction is indicated by dashed line.

Histone methylation is one of the most studied and best-characterized histone modifications that drive the regulation of complete genetic programs in the cells. However, many details of the regulation and targeting of the enzyme complexes mediating histone methylation remain elusive and a subject of debate [23]. One possible regulatory pathway is represented by the ability of certain HKMT complexes to bind different lncRNAs that serve as a targeting platform, bridging transcription factors and HKMT complexes [20,33] at the promoter regions of target genes. PRC2 is one example where it was shown by multiple experiments that it’s binding to different lncRNAs results in different physiological outcomes [34]. lncRNAs are involved in many other processes connected to histone modification and there are examples in the literature of direct interaction between lncRNAs and histone modifier complexes [4,22]. Experimental evidence supports the direct binding of WDR5, a canonical MLL complex subunit, to different lncRNAs in cells [22] indicating the involvement of lncRNAs in the regulation of MLL complexes. Taken the analogy of the PRC2, where multiple subunits are shown to be involved in lncRNA binding (Figure 4A) [15], we hypothesized that MLL proteins might also interact with lncRNAs. This hypothesis was supported by our earlier bioinformatics studies that suggested the existence of several interaction sites in the so far uncharacterized, mostly disordered regions of HKMTs [26] and our prediction presented here that the disordered segments of MLL proteins contain several putative RNA binding sequences. We chose to test the RNA binding capability of one such region of MLL4 that also contains a polyQ stretch and is affected by mutations in different cancers. As an internal control, we also tested a different region of MLL4 that contains no such predicted RNA interaction site.

Our expectation was that the isolated small regions of the MLL4 protein would bind RNAs in a nonspecific manner, such as was observed for the isolated PRC2 complex components [34]. Surprisingly, we found that MLL4_4210–4280_ bound MEG3 stronger than HOTAIR_440_ or the 50 nt random RNA, even though the determination of the exact Kd-s was not successful in all cases.

More interesting was the behavior of the MLL4_3500–3630_ region that showed dramatically different behavior with the different RNAs. Binding to MEG3 gave a Kd of 0.722 μM, while the binding to the 50 nt random RNAs proved to be so weak that a Kd calculation was not successful. Binding to HOTAIR_440_ seemed to be the strongest with an apparent Kd of 0.1 μM, but it led to the aggregation of the protein-RNA complex. The aggregation was dependent on protein-RNA ratio and could be detected through a wide protein concentration range. The same aggregation could not be observed with a shorter HOTAIR construct that consisted of 300 bases (Supplementary Figure S3). The fact that we could not induce such aggregation by the addition of MEG3, which is much longer than HOTAIR_440_, points to specific recognition rather than a side-effect of RNA length. We also observed the aggregation at low protein concentrations, but only in the presence of an appropriate amount of HOTAIR_440_, indicating that the process is not driven by the protein in itself and is not a derivative of sample preparation errors.

It has been recently revealed that many proteins can go through liquid-liquid phase separation when interacting with RNAs, leading to the formation of membraneless organelles that have a significant importance in cellular processes [35]. Experimental evidence supports the involvement of polyQ regions of proteins in the RNA mediated phase separation [28], sometimes in an RNA secondary structure-dependent manner [36]. Since MLL4_3500–3630_ sequence contains 22.9% glutamine residues and a continuous run of 15 glutamines (Figure 1A), it is not unfounded to speculate that this specific region plays a role in the observed anomaly but the fact that it only occurs with one of the tested RNA constructs, indicates that the process is coordinated by the RNA itself. One possibility is that the longer HOTAIR construct contains more than one binding sites for MLL4_3500–3630_, thus facilitating the formation of higher order protein-RNA structures. Alternatively, HOTAIR_440_ may have the ability to form secondary structures not found in HOTAIR_300_ or MEG3, which would also provide an explanation for the different behavior of the three systems. As MLL4 is the only HKMT that contains long polyglutamine repeat stretches [26], phase separation might be a regulatory step specific for this protein. Therefore, it is certainly promising to investigate this peculiar phenomenon in more detail.

Since both tested lncRNAs are implicated in different cancers [5,37,38] involving leukemias, our finding that MLL4 has a capacity to bind them raises the possibility that lncRNAs play a role in MLL/COMPASS complex targeting and regulation to a larger extent than currently recognized.

Although cellular experiments are necessary to prove the validity of the observed interactions, our findings provide the first insights into the structure and function of two regions of MLL4 that have been uncharacterized so far. We were able to show that these regions are capable of RNA binding and may be involved in the lncRNA mediated regulation of the MLL4 complexes. Based on our results, we suggest that and MLL4 complexes utilize different regions on their surface to bind lncRNAs (Figure 4B), similarly to the way PRC2 subunits take part in lncRNA binding. As it was shown that lncRNA binding to WDR5 increases the dwelling time of the protein on the chromatin surface [22], binding of the same RNA to MLL4 might facilitate and accelerate the assembly of a functional methyltransferase complex. Since lncRNAs are large molecules that can adopt various secondary structures and interact with many different partners simultaneously, it is plausible to speculate that a specific and high-affinity interaction can be achieved by the combination of different binding sites distributed along the large surfaces of multi-subunit complexes. Given the central role of histone modifications in gene regulation, it is essential to understand the mechanisms that regulate this process. Mounting evidence supports the involvement of lncRNAs in the coordination of histone modifying enzymes but the exact molecular details of their interactions with proteins are yet to be discovered. Recognizing the importance of the disordered/structurally uncharacterized regions of HKMTs in these interactions might be the first step towards a more complete picture regarding the regulation of histone methylation.

## 4. Materials and Methods

### 4.1. Bioinformatics Analysis

Disorder and disordered binding site predictions were performed with the IUPred2A online prediction tool (https://iupred2a.elte.hu/) [39] which incorporates the IUPred and Anchor predictors. RNA binding regions located in disordered regions were predicted using the DisoRDPbind tool (http://biomine.cs.vcu.edu/servers/DisoRDPbind/) [40]. Cancer-related single nucleotide polymorphisms in the long conserved IDR regions were collected from the BioMuta v2.0 [41] and COSMIC databases [42].

### 4.2. Accession Numbers

HOTAIR: Gene ID: 100124700

MEG3: Gene ID: 55384

MLL1: Uniprot: Q03164

MLL2: Uniprot: Q9UMN6

MLL3: Uniprot: Q8NEZ4

MLL4: Uniprot: O14686

### 4.3. Overexpression and Purification of MLL4 Protein Regions

The same methods of protein overexpression and purification were used for both protein constructs, MLL4_3500–3630_ and MLL4_4210–4280_. DNA sequences coding for each protein were cloned into pET22b cloning vector. Induction was done for 4 h at 28 °C by 0.1 M IPTG, cells were pelleted by centrifugation (4000 rpm, 20 min, 4 °C) then lysed by sonication in lysis buffer (50 mM Tris, 200 mM NaCl, 0.5% Triton X-100 pH 8.0 and EDTA-free SIGMAFAST Protease Inhibitor Cocktail Tablets), cell debris was removed by centrifugation (12,100 rpm, 40 min, 4 °C). The supernatant was filtered through 0.2 μm nitrocellulose filter then purified over HisTrap HP column on an AKTA Explorer system using a gradient elution of two buffers (Buffer A: 20 mM imidazole, 200 mM NaCl, 20 mM Tris. pH 7.5. Buffer B: 1 M imidazole, 200 mM NaCl, 20 mM Tris, pH 7.5). Representative purification results are shown on Supplementary Figure S7. The mostly disordered nature of the MLL4_4210–4280_ region was highlighted by its appearance at a larger size than its actual molecular weight (17 kDa vs. 7 kDa). Elution fractions containing sufficiently pure proteins were dialyzed against distilled water then lyophilized and stored at −20 °C. Lyophilized proteins were dissolved before use in ultrapure water or the appropriate assay buffer. The identity of the purified proteins was confirmed by mass spectrometry.

### 4.4. RNA Preparation

HOX transcript antisense RNA (HOTAIR):

HOTAIR_300_ (140–440 nt) and HOTAIR_440_ (1–440 nt) DNA sequences cloned into pEX-A128 vector were purchased from Eurofins Genomics (Ebersberg, Germany). After 2 h digestion with EcoRV restriction enzyme at 37 °C, the gel-purified, linearized DNA templates were used to synthesize RNA by in-vitro transcription.

Maternally Expressed 3 (MEG3) lncRNA:

pCI-MEG3 was a gift from Anne Klibanski (Addgene plasmid #44727, Watertown, MA, USA) [43]. Primers to obtain the DNA template for in vitro transcription were as follows:

T7 RNA promoter region followed by:

MEG3 forward primer:

TAATACGACTCACTATAGGGGCAGAGAGGGAGCGCGCCTTGG

MEG3 reverse primer:

GATATCTTTTTGTTAAGACAGGAAACACATTTATTGAGAGC

50 nt RNA:

50 nucleotide RNA was an artificial randomized RNA sequence.

DNA templates were T7 promoter region followed by:

50 nt forward oligo:

TAATACGACTCACTATAGAAGAATGGCCTCGCGGAGGCATGCGTCATGCTAGCGTGCGGGGTACTCTT and

50 nt reverse oligo:

AAGAGTACCCCGCACGCTAGCATGACGCATGCCTCCGCGAGGCCATTCTTCTATAGTGAGTCGTATTA

Transcribed RNA:

GAAGAAUGGCCUCGCGGAGGCAUGCGUCAUGCUAGCGUGCGGGGUACUCUU

All primers and oligonucleotides were purchased from Sigma-Aldrich Ltd. (St. Louis, MO, USA).

Tested RNAs were synthesized by in vitro transcription carried out with New England BioLabs HiScribe™ T7 Quick High Yield RNA Synthesis Kit (Ipswich, MA, USA). Fluorescein-labelled, single-stranded RNA probes were generated by using Roche (Basel, Switzerland) Fluorescein RNA Labeling Mix (11685619910) and NEB 10× T7 reaction buffer (#B2041A). After transcription, remaining DNA templates were eliminated with DNaseI treatment. RNA sample purification was carried out using Macherey-Nagel NucleoSpin^®^ RNA Clean-up XS Kit (Düren, Germany). The quality and intactness of the purified transcription products were analysed by native and formaldehyde agarose gel electrophoresis.

Biotinylation of the RNAs was performed using Pierce™ RNA 3′ End Biotinylation Kit (Cat. Number 20160, Thermo Fisher Scientific, Waltham, UK) according to the instructions of the manufacturer. Overnight incubation at 16 °C was applied for the ligation of the biotin label. Final RNA concentrations were determined using NanoDrop™ 1000 Spectrophotometer (Thermo Fisher Scientific, Waltham, UK).

Purified RNAs were stored −80 °C until usage in the presence of RNAINH-RO Roche Protector RNase Inhibitor (20U).

### 4.5. Far-UV CD Measurements

CD measurements were performed in quartz cells of 0.1 mm pathlengths using a Jasco J-810 (Jasco, Tokyo, Japan) spectropolarimeter. Far-UV CD spectra were recorded in the range of 180–260 nm with a scanning speed of 20 nm/min, bandwidth of 1 nm and integration time of 4 s. 6 scans were accumulated. Thermal denaturation was recorded in a 1 mm cell at 220 nm from 10 to 100 °C with scanning rate of 120 °C/h. The temperature was controlled using a PTE Peltier unit. The thermal denaturation profile was fitted according to the Gibbs-Helmholtz equation assuming a two-state model, which is represented by a sigmoidal curve [44]. CD spectra were quantitatively analyzed by the BeStSel method [29,30] (http://bestsel.elte.hu).

### 4.6. Microscale Thermophoresis

RNA-protein binding assays were carried out on a Microscale Thermophoresis system (Monolith NT. 115 from NanoTemper Technologies, München, Germany). Standard treated capillaries (Cat. Number: MO-K002) were used for measurements. Instrument settings are presented in Table 2.

Normalized fluorescence values after 1.25 s after turning on the IR laser were used as T-jump values.

RNA concentrations were set to give an initial raw fluorescence between 300 and 1000 counts and varied between 30 and 100 nM. All experiments were done at room temperature. DEPC-treated PBS buffer containing 0.05% NP-40 was used as assay buffer.

### 4.7. Electrophoretic Mobility Shift Assay (EMSA)

LightShift^®^ Chemiluminescent RNA EMSA Kit (Thermo Scientific, Cat. No. 20158, Thermo Fisher Scientific, Waltham, UK) was used for the EMSA experiments. Assay control was performed according to the instructions of the manufacturer with the control reaction provided with the kit. In short, 6.25 nM biotin-labeled IRE RNA was incubated with 2 μg of cytosolic liver extract with or without 1 μM of unlabeled IRE RNA. The result of the assay control is presented on Supplementary Figure S6. Binding, electrophoresis and detection of the tested RNAs with the proteins were carried out following the protocol of the kit. Briefly, proteins of varying concentrations were incubated with 1 or 2 nM of RNAs for 30 min at room temperature, then loaded on 4 or 6% native polyacrylamide gels. RNA was transferred to nitrocellulose membranes using Trans-Blot^®^ Turbo™ Transfer System (Bio-Rad, Hercules, CA, USA) and crosslinked to the membrane by UV-light crosslinking. After proper washing and blocking, biotin labeled RNA was detected by chemiluminescence using Streptavidin-Horseradish Peroxidase Conjugate.

## Figures and Tables

**Table 1:** Predicted RNA binding regions in the disordered regions of Mixed Lineage Leukemia (MLL) proteins (aa positions).

MLL1	MLL2	MLL3	MLL4
296–327	84–107	1068–1079	1559–1567
348–408	184–234	1678–1695	3526–3581
415–418	241–244	1701–1709	3899–3983
1155–1194	536–560	1715–1737	4960–5014
1977–1992	783–806	2406–2409	5147–5165
3854–3861	820–828	3052–3073	5227–5251
	1753–1778	3246–3250	
	2600–2616	3394–3427	
	2685–2709	4330–4356	
		4514–4524	
		4586–4625	

**Table 2:** **Instrument settings for MST**.

Title	LED Power (%)	MST Power (%)	Before MST (s)	MST on (s)	After MST (s)	Delay (s)
Round 1	10 or 40	20	5	30	5	25
Round 2	10 or 40	40	5	30	5	25

## Supplementary Materials

The following are available online at http://www.mdpi.com/1422-0067/19/11/3478/s1.

## Abbreviations

EMSA	Electrophoretic mobility shift assay
EZH2	Enhancer of zeste homolog 2
HKMT	Histone lysin methyltransferase
HOTAIR	HOX transcript antisense RNA
lncRNA	Long non-coding RNA
MEG3	Maternally Expressed 3
MLL	Mixed lineage leukemia
MST	Microscale thermophoresis
PRC2	Polycomb repressive complex
WDR5	WD repeat-containing protein 5
