# Supplementary material for: Disordered Regions of Mixed Lineage Leukemia 4 (MLL4) Protein Are Capable of RNA Binding
Source: Int J Mol Sci. 2018 Nov 5;19(11):3478. doi: 10.3390/ijms19113478 (PMC6274713; doi:10.3390/ijms19113478)

Supplementary figure 1

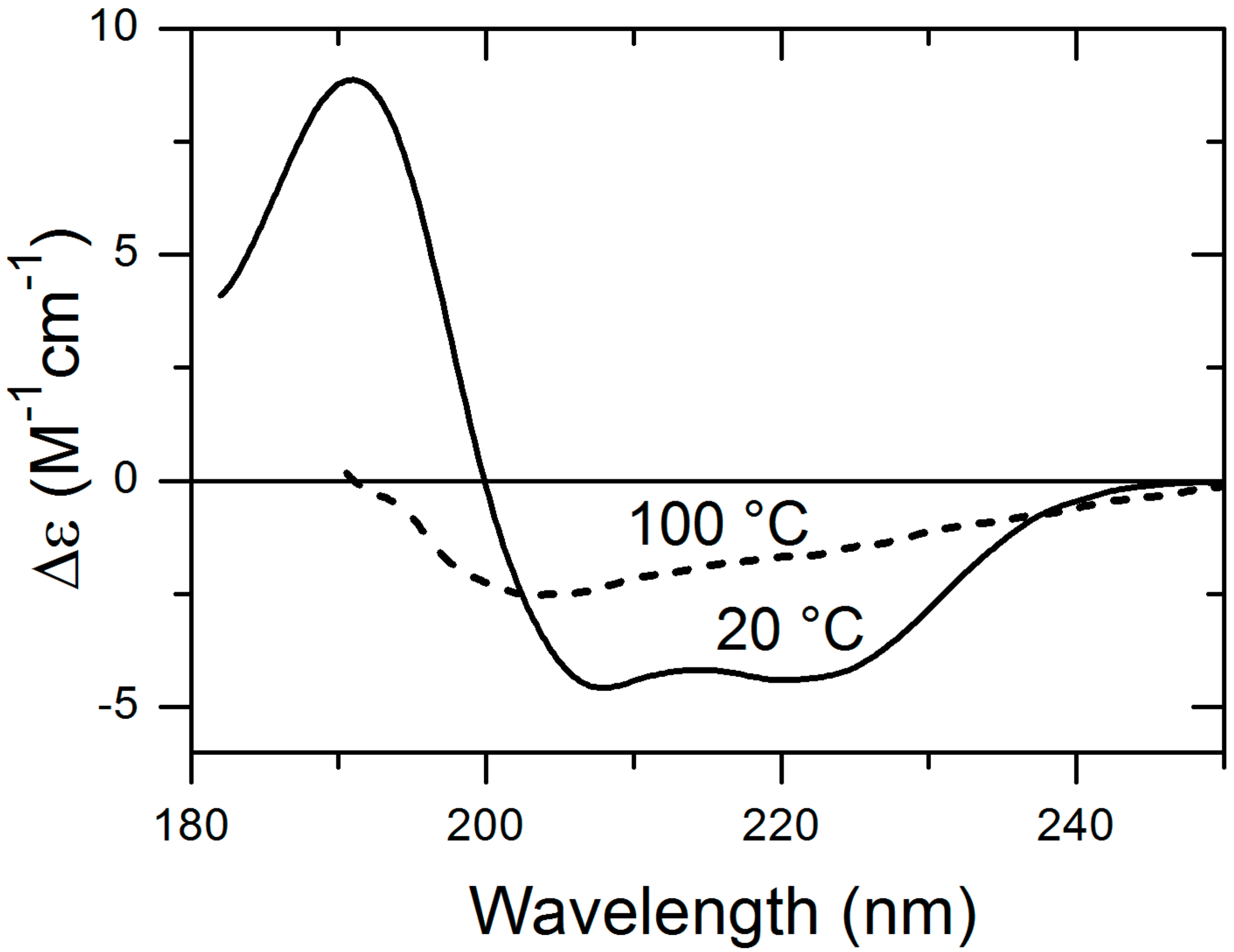

Supplementary figure 2

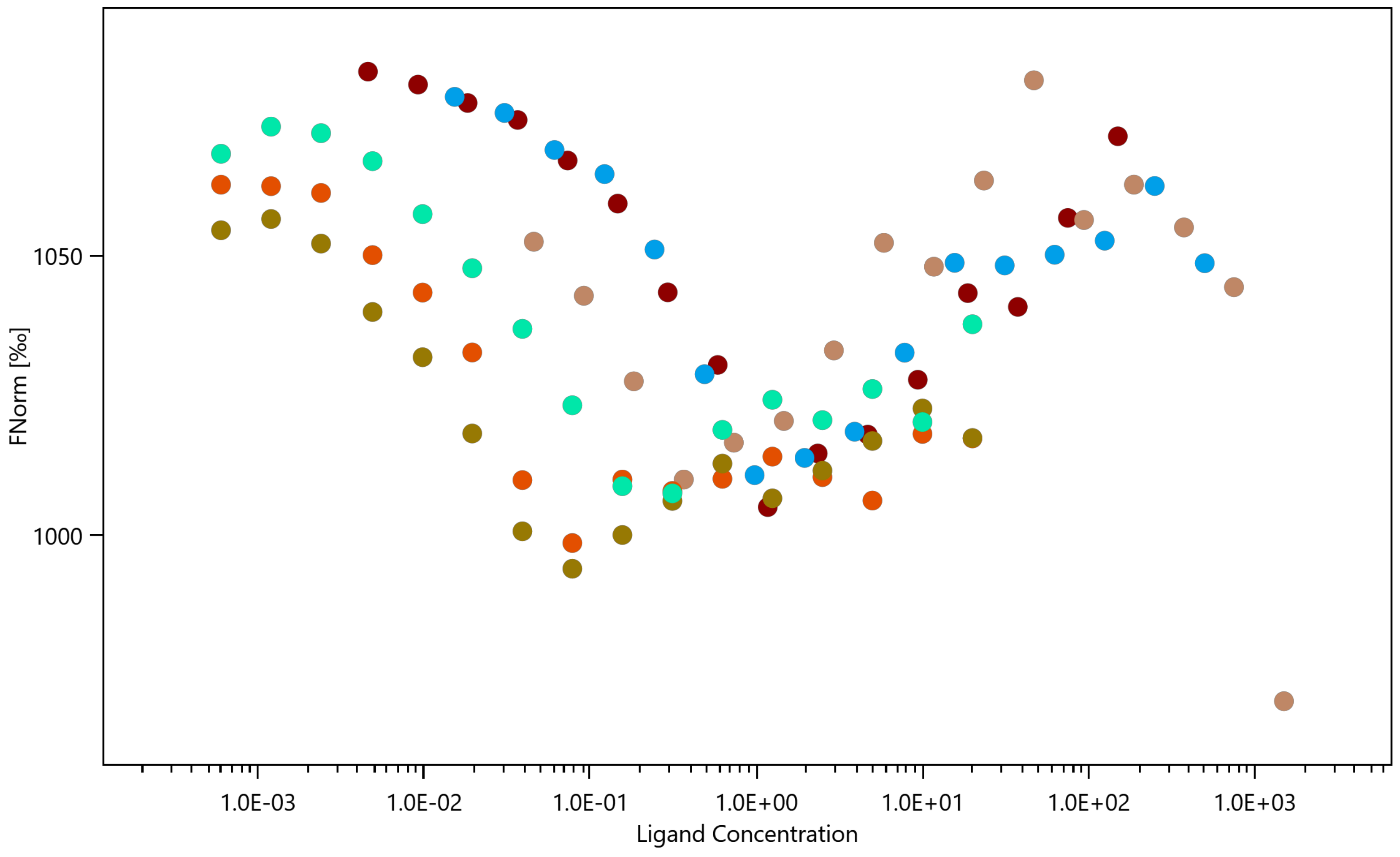

Supplementary figure 3

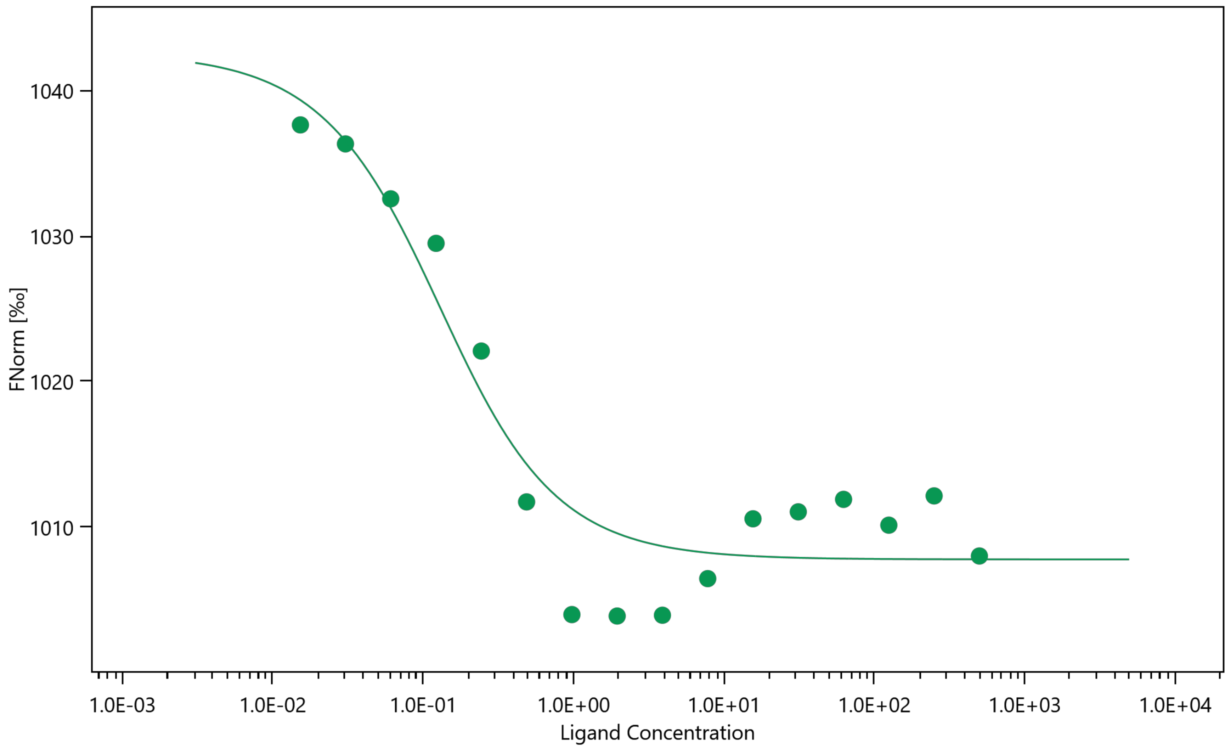

Supplementary figure 4

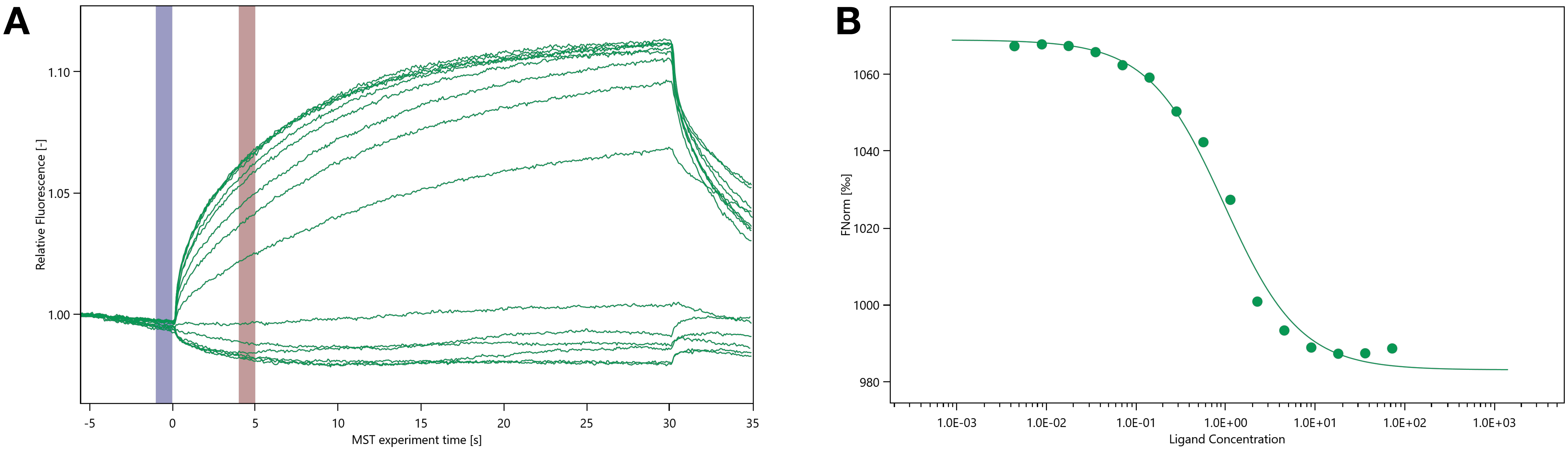

Supplementary figure 5

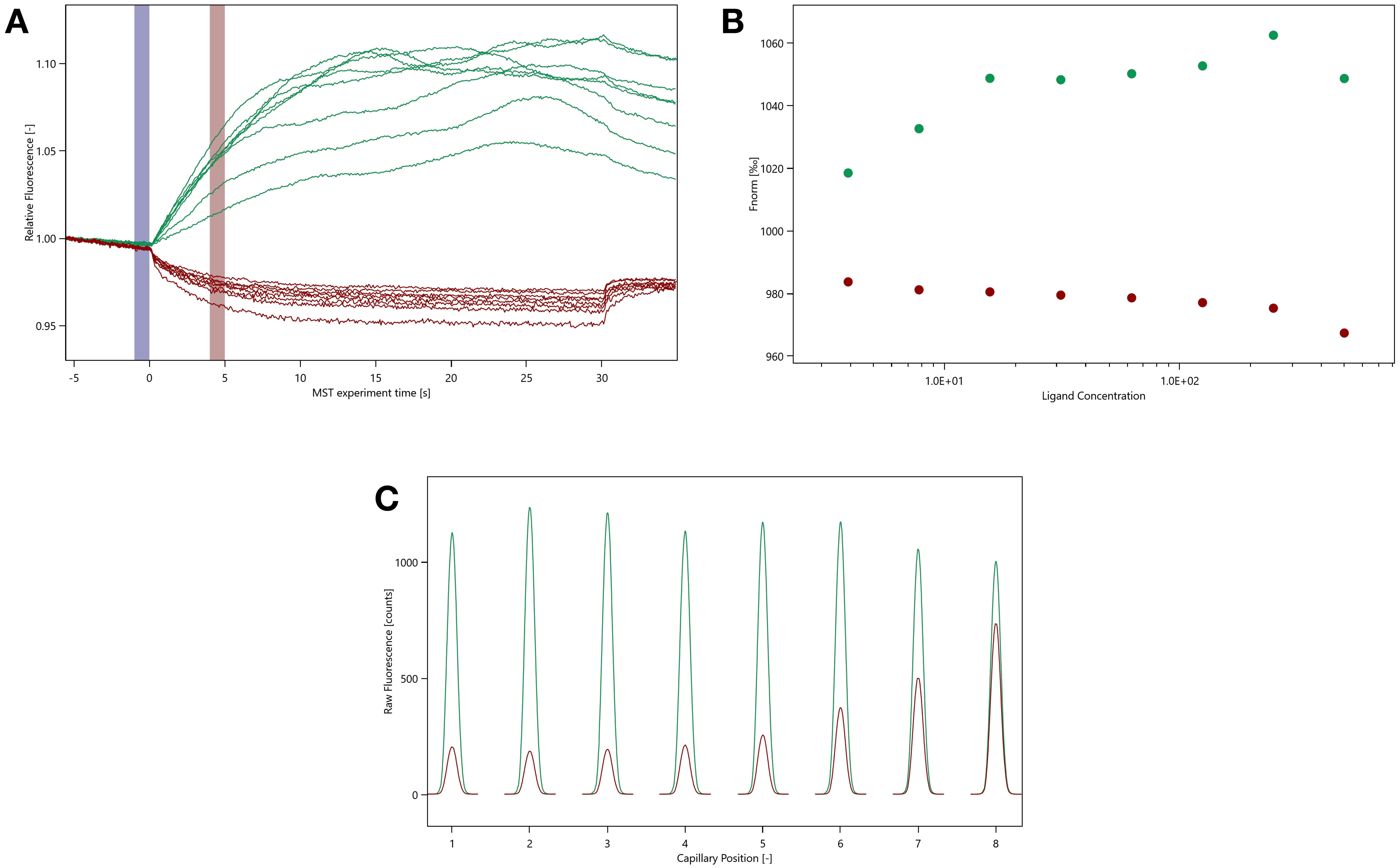

Supplementary figure 6

Cytosolic liver extract (μg)	0	2	2
Biotin-IRE RNA (nM)	6.25	6.25	6.25
Unlabeled IRE RNA (μM)	0	0	1

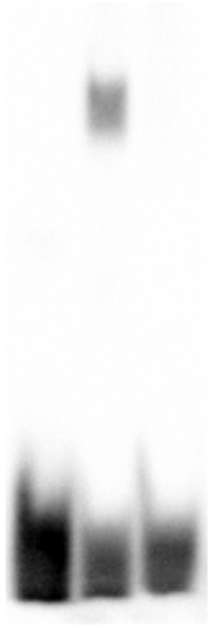

Supplementary figure 7

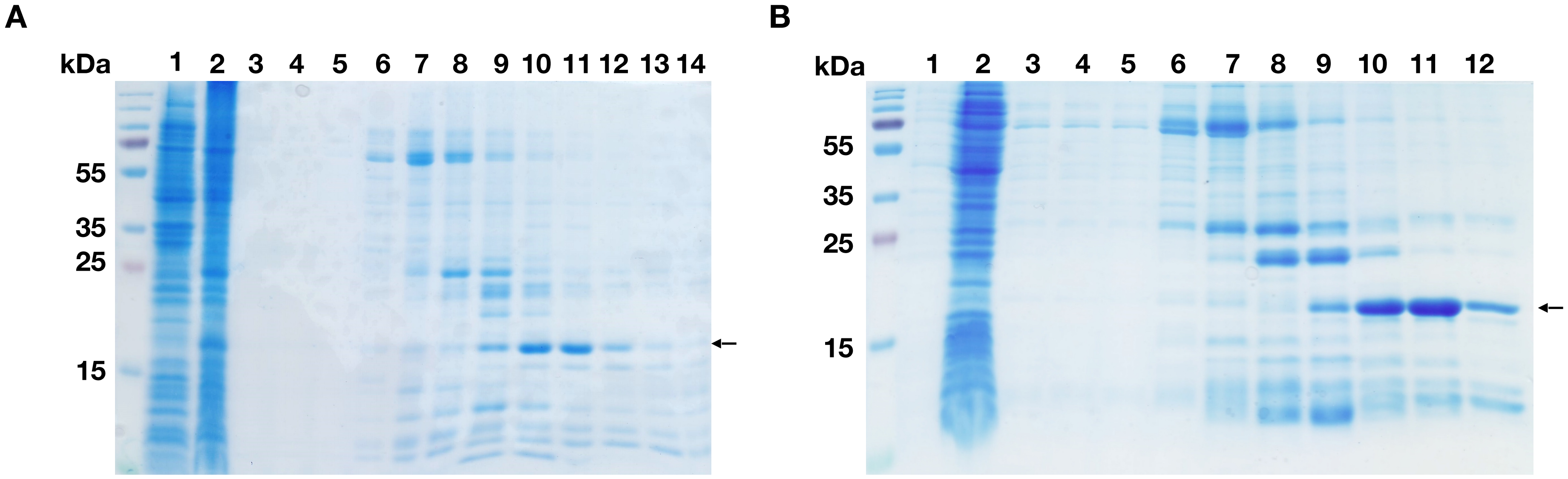

Supplement: Supplementary file 1 [file ijms-19-03478-s001.pdf]
